# A Multidimensional Evaluation of Sakuranetin against *Schistosoma mansoni*: From Drug-Likeness to *In Vivo* Efficacy

**DOI:** 10.1021/acsomega.6c03820

**Published:** 2026-06-12

**Authors:** Dalete Christine S. Souza, Pedro Enrico H. Tesser, Erica Fernanda da S. Tirelli, Ruqaya Jasim, Monique C. Amaro, Rayssa A. Cajas, Dion R. Brocks, Josué de Moraes, João Henrique G. Lago

**Affiliations:** † Center for Natural and Human Sciences, Federal University of the ABC, São Paulo 09210-180, SP Brazil; ‡ Katz Group Centre for Research, 3158 University of Alberta, Edmonton, Alberta T6G 2G5, Canada; § Center for Research on Neglected Diseases, 92928 Guarulhos University, São Paulo 07023-070, Brazil; ∥ Centre for Research on Neglected Diseases, Brazil University, São Paulo 08230-030, Brazil

## Abstract

Schistosomiasis is a highly prevalent neglected tropical disease affecting more than 250 million people worldwide and currently relies on praziquantel (PZQ) as the only available treatment. This dependence raises concerns about the long-term sustainability of current control strategies and underscores the urgent need for new anthelmintic agents. In this context, natural products represent an important source of novel bioactive compounds. In the present study, the antischistosomal potential of sakuranetin (SAK), a flavanone isolated from *Baccharis lateralis* (Asteraceae), was evaluated. *In silico* analysis indicated favorable drug-like properties, including high predicted gastrointestinal absorption and absence of structural alerts. *In vitro* and *in vivo* safety assessments showed no cytotoxicity in Vero cells (CC_50_ > 500 μM) and no acute toxicity in *Caenorhabditis elegans* (LC_50_ > 1000 μM). When evaluated for direct antiparasitic effects, SAK did not exhibit detectable *in vitro* activity against adult *Schistosoma mansoni* worms. However, oral administration of SAK (400 mg/kg) in a *S. mansoni* murine model significantly reduced total worm burden by 84.4% and decreased fecal egg burden by 87.0% (PZQ: 88.2 and 95.9%, respectively). To support pharmacokinetic studies, a sensitive HPLC-UV method was validated (LLOQ = 50 ng/mL). SAK exhibited high plasma protein binding (unbound fraction = 8.3%) and was predominantly metabolized via glucuronidation in rat liver microsomes (*t*_1/2_ = 149 min). These findings support SAK as a promising natural product-derived lead with significant *in vivo* antischistosomal activity and favorable drug-like properties.

## Graphical abstract

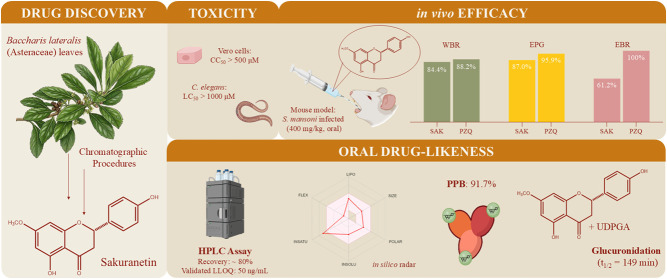

## Introduction

Neglected Tropical Diseases (NTDs) affect over one billion people worldwide, primarily in tropical and subtropical regions, where they contribute to persistent cycles of poverty through chronic illness, disability, and social stigma.^1,2^ Although global health initiatives have advanced in recent years, NTDs continue to receive limited financial support and have historically been neglected in research and drug development efforts.^3^ In 2020, the World Health Organization (WHO) introduced a roadmap aligned with the Sustainable Development Goals (SDGs) aimed at the control, elimination, or eradication of NTDs by 2030.^4^ Nevertheless, insufficient investment, limited therapeutic innovation, and the lack of effective treatments remain major obstacles to achieving these objectives.^5,6^

Among the neglected tropical diseases, schistosomiasis remains one of the most prevalent parasitic infections worldwide. Caused by blood flukes of the genus *Schistosoma*, the disease affects more than 250 million people globally. Current chemotherapy depends exclusively on praziquantel (PZQ), a drug that has been widely used since the 1980s.^7^ Dependence on a single therapeutic agent for such a large at-risk population represents a major public health concern, especially considering the limited efficacy of praziquantel against immature parasites and the potential emergence of drug resistance over time.^8,9^ Together, these limitations reinforce the urgent need for the discovery and development of new antischistosomal therapies.

Natural products constitute an important source of bioactive molecules, with nearly half of currently approved drugs being derived from or inspired by compounds of natural origin, including those obtained from plants, microorganisms, and marine organisms.^10^ Within this context, species of the genus *Baccharis* (Asteraceae) have long been used in traditional medicine due to their anti-inflammatory, antioxidant, and antiparasitic properties.^11^ Notably, extracts and metabolites isolated from *Baccharis* species, such as *Baccharis lateralis* and *Bothrops matogrosensis*, have shown promising *in vitro* and *in vivo* activity against *Schistosoma mansoni*,^12,13^ highlighting their potential as sources of new anthelmintic agents. In particular, the Brazilian species *B. lateralis* is known to be rich in flavonoids and other bioactive metabolites with relevant pharmacological potential.^14^

Sakuranetin (SAK) is a naturally occurring flavanone found in several plant families, including Asteraceae. Previous studies have demonstrated a wide spectrum of biological activities for this compound, including antimutagenic, anticancer, antiviral, anti-inflammatory, antidiabetic, antimicrobial, and antiprotozoal effects.^15,16^ Regarding parasitic diseases, SAK has shown promising activity against *Trypanosoma cruzi* and *Leishmania* spp., with reported IC_50_ values of 20.17 and 43–52 μg/mL, respectively.^17,18^ Nevertheless, its potential activity against *S. mansoni* remains unexplored. Therefore, the present study investigated the antischistosomal potential of SAK through an integrated approach involving *in silico* drug-likeness analysis, *in vivo* efficacy evaluation using a murine model of schistosomiasis, and HPLC-based studies focused on plasma protein binding and microsomal glucuronidation.

## Results

### Chemical Characterization

The ^1^H and ^13^C NMR as well as ESI-HRMS data (Figures S1–S4, Supporting Information) were consistent with those previously reported in the literature,^18^ allowing the identification of the isolated compound as sakuranetin (SAK) (Figure 1a). HPLC analysis indicated a purity of 99% for SAK.

**Figure 1:**
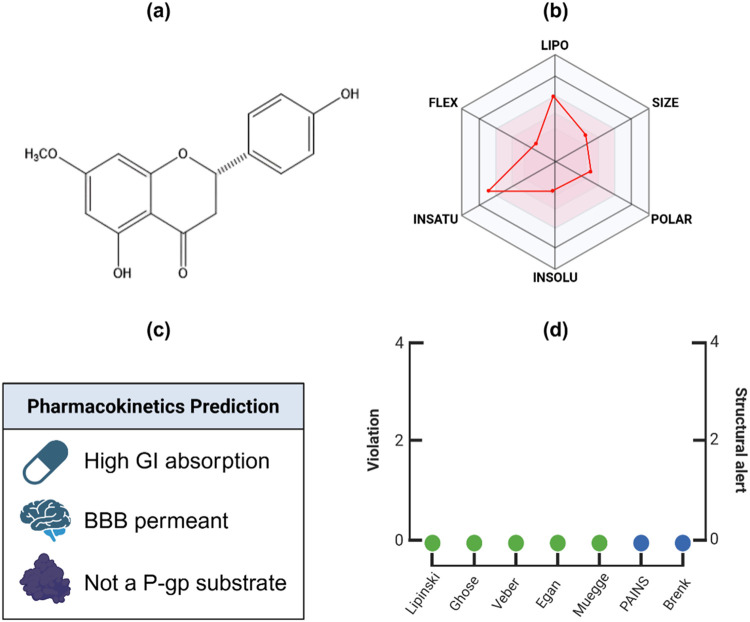
*In silico* drug-likeness and pharmacokinetic profile of SAK. (a) Chemical structure of sakuranetin (SAK). (b) SwissADME bioavailability radar illustrating the physicochemical properties of SAK in relation to the optimal oral bioavailability range (shaded region), including lipophilicity (LIPO), molecular size (SIZE), polarity (POLAR), solubility (INSOLU), flexibility (FLEX), and saturation (INSATU). (c) Predicted pharmacokinetic parameters indicating high gastrointestinal (GI) absorption, blood–brain barrier (BBB) permeability, and lack of P-glycoprotein (P-gp) substrate liability. (d) Drug-likeness and medicinal chemistry assessment demonstrating compliance with major drug-likeness rules (Lipinski, Ghose, Veber, Egan, and Muegge) and absence of structural alerts according to PAINS and Brenk filters.^19^

### *In Silico* Drug Likeness

The physicochemical and pharmacokinetic properties of SAK were predicted using the SwissADME platform.^19^ According to the bioavailability radar (Figure 1b), SAK exceeded only one of the six evaluated parameters, namely unsaturation (INSATU), while the remaining properties—including polarity (TPSA), molecular size, flexibility (FLEX), lipophilicity (LIPO), and solubility (INSOLU)—remained within the recommended range for oral bioavailability. A more comprehensive description of these parameters is provided in Table S1 (Supporting Information), which summarizes data related to physicochemical properties, lipophilicity, water solubility, pharmacokinetics, drug-likeness, and medicinal chemistry.

### *In* *Vitro* Antiparasitic Activity and Toxicity

SAK did not exhibit cytotoxic effects in Vero cells at concentrations up to 500 μM (CC_50_ > 500 μM). Likewise, no signs of acute toxicity were observed in *Caenorhabditis elegans*, with nematode survival maintained at concentrations up to 1000 μM (LC_50_ > 1000 μM). These findings indicate a favorable safety profile for SAK in both mammalian cells and the nonparasitic nematode *C. elegans*. In contrast, SAK showed no detectable *in vitro* antiparasitic activity against adult *S. mansoni* worms at concentrations up to 50 μM (EC_50_ > 50 μM). A summary of the antiparasitic and cytotoxicity results is presented in Table S3.

### *In Vivo* Antischistosomal Activity

Oral administration of SAK (400 mg/kg) to *S. mansoni*-infected mice resulted in a significant reduction of total worm burden (84.4%) compared with untreated animals (Figure 2). This effect was comparable to that observed for praziquantel (PZQ, 400 mg/kg), which reduced worm burden by 88.2%. Furthermore, SAK treatment markedly decreased fecal egg burden by 87.0% (Figure 3), while PZQ achieved a 95.9% reduction.

**Figure 2:**
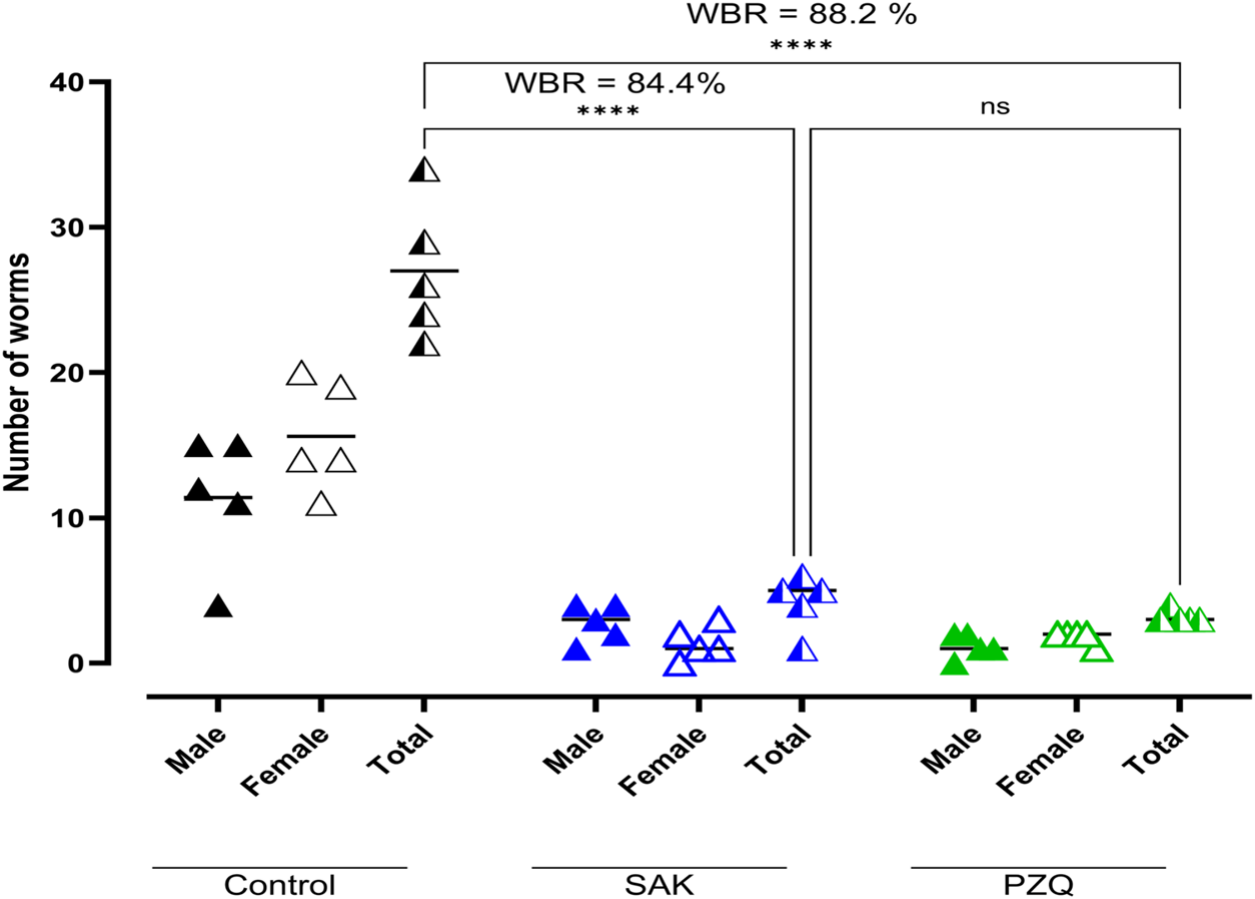
Effect of SAK on worm burden reduction (WBR) in mice infected with *S. mansoni*. SAK was administered orally as a single dose (400 mg/kg) 49 days after infection to mice harboring adult *S. mansoni* worms. Animals were euthanized on day 56 postinfection, and parasite burden was determined according to worm sex (male and female). Each point represents an individual animal (*n* = 5 per experimental group), and horizontal bars indicate median values. Praziquantel (PZQ, 400 mg/kg) was used as the reference drug. Statistical significance: *p* < 0.0001.

**Figure 3:**
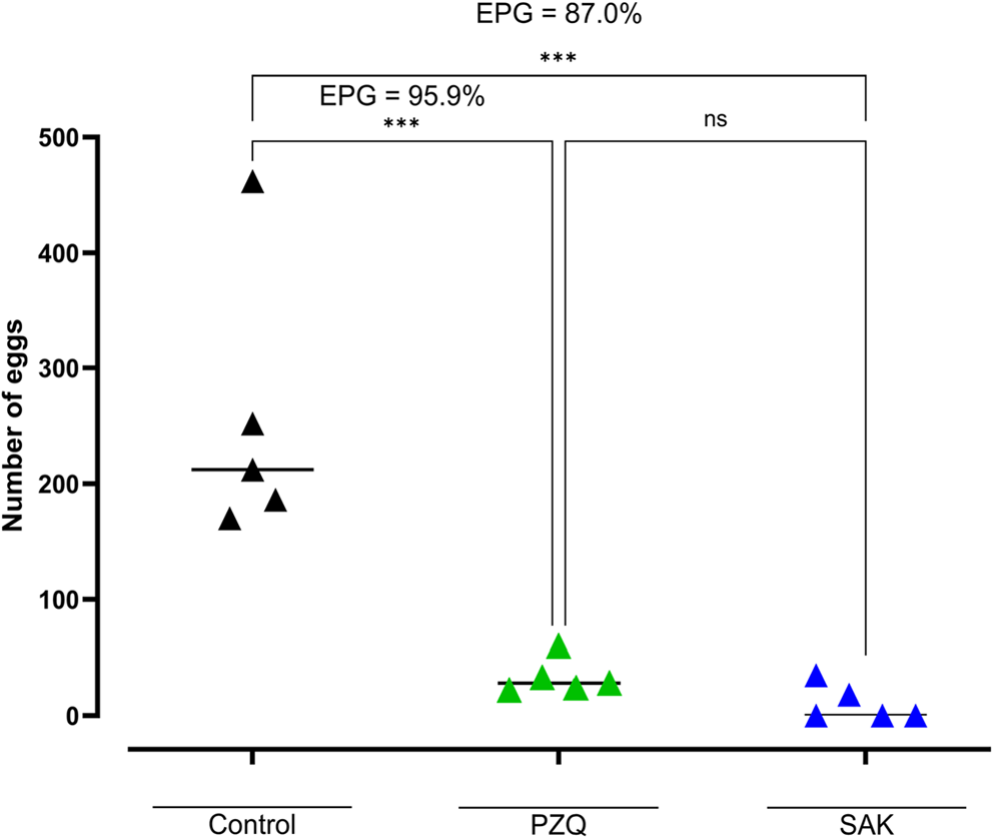
Effect of SAK on fecal egg burden in mice infected with *S. mansoni*. SAK was administered orally as a single dose (400 mg/kg) 49 days after infection to mice harboring adult *S. mansoni* worms. On day 56 postinfection, all animals were euthanized, and fecal egg counts were determined using the Kato–Katz technique. Each point represents an individual animal (*n* = 5 per group), while horizontal bars indicate median values. Praziquantel (PZQ, 400 mg/kg) was used as the reference drug. Statistical significance: *p* < 0.001.

### Assay Performance

HPLC analysis revealed retention times of 4.94 and 16.14 min for quercetin (QUE, internal standard) and sakuranetin (SAK), respectively (Figure S5, Supporting Information). The chromatograms exhibited symmetrical and well-resolved peaks for both SAK and QUE, without interference from endogenous plasma components. Mean recovery values were approximately 80% for SAK and 60% for QUE. The method demonstrated excellent linearity between peak area ratios (drug/IS) and plasma concentrations, with correlation coefficients (*r* ^2^) greater than 0.99 over the concentration range of 50–10,000 ng/mL using 0.1 mL plasma samples. Method validation also showed satisfactory precision and accuracy, with intra- and interday coefficients of variation (CV%) below 22% for all evaluated concentrations (Table 1).

**Table 1:** Validation Data for the Quantification of SAK in Rat Plasma (*n* = 5 per Concentration)^a^

	intraday (ng/mL)			interday (ng/mL)
nominal (ng/mL)	mean + SD [mean error] (CV%)			mean + SD [mean error] (CV%)
50	53 ± 5	44 ± 2	51 ± 5	49 ± 4
	[5.37] (9.38)	[−12.36] (4.60)	[1.81] (9.84)	[−1.73] (7.80)
250	280 ± 11	227 ± 31	238 ± 18	251 ± 27
	[−15.21] (3.68)	[−9.06] (13.62)	[−4.95] (7.39)	[0.40] (10.57)
1000	921 ± 58	980 ± 294	1076 ± 37	992 ± 64
	[−7.85] (6.29)	[−2.02] (30.01)	[7.58] (3.46)	[−0.76] (6.41)
2500	2366 ± 86	2434 ± 119	2404 ± 127	2401 ± 28
	[−5.36] (3.63)	[−2.62] (4.91)	[−3.85] (5.30)	[−3.95] (1.17)
5000	4867 ± 274	5037 ± 218	4799 ± 247	4901 ± 100
	[−2.66] (5.63)	[0.75] (4.33)	[−4.03] (5.15)	[−1.98] (2.05)
10,000	10,113 ± 627	10,018 ± 345	10,146 ± 659	10,093 ± 5
	[1.13] (6.20)	[0.18] (3.45)	[1.46] (6.50)	[0.93] (0.54)

a An independent calibration curve was prepared daily and used to generate the standard curve for quantification of SAK concentrations in plasma samples. Analyses were performed on three independent days to determine intra- and inter-day precision and accuracy. Precision was expressed as the coefficient of variation (CV%), while accuracy was evaluated as the mean percentage error between measured and nominal concentrations.

These parameters did not meet the acceptable validation criteria for concentrations below 50 ng/mL when using 0.1 mL plasma samples. No detectable peaks were observed in blank plasma samples containing neither SAK nor internal standard (IS) when injected immediately after samples containing 10,000 ng/mL of SAK and IS, confirming the absence of carryover in the analytical system. The stability of SAK under different time and temperature conditions was also evaluated (Table S2, Supporting Information). Percentage deviations remained below 20% for the lower concentration (500 ng/mL) and below 15% for the higher concentration (7500 ng/mL), demonstrating acceptable stability under the tested conditions.

### Plasma Protein Binding

In rat plasma, SAK exhibited a low unbound fraction of 8.3 ± 0.1% at a spiked concentration of 10,000 ng/mL, indicating extensive plasma protein binding.

### Glucuronidation of SAK by Rat Liver Microsomes

The metabolic stability of SAK was assessed by monitoring the depletion of the parent compound at predetermined incubation intervals. SAK exhibited a half-life (*t*_1/2_) of 149 ± 8 min and an apparent intrinsic clearance (CL_int_) of 4.7 ± 0.2 μL/min/mg protein. After 2 h of incubation, approximately 46% of the initial amount of SAK had been metabolized, presumably through glucuronidation.^20^ Representative chromatograms obtained from the microsomal assay at different incubation times (male rat 1) are presented in Figure S7 (Supporting Information).

## Discussion

The development of new therapeutic alternatives for schistosomiasis remains a major priority due to the exclusive dependence on praziquantel (PZQ) and concerns regarding the long-term effectiveness of monotherapy for a disease affecting more than 250 million people worldwide. In the present study, *in silico*, *in vitro*, and *in vivo* approaches were combined to investigate the pharmacological profile of sakuranetin (SAK), a flavonoid isolated from *B. lateralis*.

Although biological evaluations are typically initiated with *in vitro* assays followed by *in vivo* studies, the biotransformation of compounds and their ADME properties (absorption, distribution, metabolism, and excretion) can substantially influence biological responses under physiological conditions.^20^ In this context, the absence of detectable *in vitro* activity contrasts with the pronounced *in vivo* efficacy observed for SAK, highlighting the limitations of conventional parasite-based assays. Such assays fail to account for host-dependent factors, including metabolism, systemic exposure, and interactions with blood components, which are particularly important for intravascular parasites such as *S. mansoni*. For this reason, an ADME-based approach was employed to compare the pharmacokinetic profile of SAK with those of compounds commonly used for oral administration. *In silico* pharmacological analyses performed using the SwissADME platform (Figure 1b and Table S1, Supporting Information) revealed no violations of major drug-likeness criteria, including Lipinski’s rules and PAINS filters. In addition, the predicted high gastrointestinal (GI) absorption suggests favorable oral bioavailability, reflecting the ability of SAK to efficiently cross the intestinal barrier. Regarding cytochrome P450 interactions, SAK was predicted to inhibit CYP1A2, CYP2C19, and CYP3A4, while showing no inhibitory potential toward CYP2C9 and CYP2D6 (Figure 1d and Table S1, Supporting Information). Nevertheless, previous studies have demonstrated that the inhibitory effects of SAK on cytochrome P450 enzymes do not exceed 20%.^20^

When evaluated *in vivo*, SAK exhibited pronounced antischistosomal activity, reducing the *S. mansoni* worm burden by 84.4%, a result comparable to that achieved with PZQ administered at the same oral dose (Figure 2). This efficacy is particularly noteworthy when compared with other flavonoids previously reported to display *in vivo* antischistosomal activity, such as hesperidin and kaempferol, which reduced worm burden by 47.5 and 25.5%, respectively.^12,21^ In addition, SAK significantly decreased fecal egg burden by 87.0% (Figure 3), approaching the reduction observed for PZQ (95.9%) and surpassing those reported for hesperidin (58.6%) and kaempferol (28.8%). This effect is especially relevant, as egg production and viability are key factors driving both disease pathology and parasite transmission in schistosomiasis.^22,23^ In light of these findings, although the precise mechanism of action of sakuranetin against *S. mansoni* has not yet been fully elucidated, current evidence suggests a multifactorial mode of action. Previous studies have demonstrated that SAK undergoes metabolic biotransformation into bioactive flavonoids such as naringenin and eriodictyol, which may contribute to its pharmacological effects.^24^ In addition, SAK has been reported to modulate oxidative stress and inflammatory pathways, including the regulation of antioxidant enzymes and NF-κB signaling in mammalian models,^25^ indicating that host-mediated responses may play an important role in its observed *in vivo* efficacy. Supporting this hypothesis, SAK exhibited no detectable cytotoxicity toward mammalian cells (CC_50_ > 500 μM in Vero cells) and showed no acute toxicity in *C. elegans* (LC_50_ > 1000 μM). Together, these findings indicate a favorable safety profile and reinforce the potential of SAK as a selective antischistosomal candidate. To support future pharmacokinetic investigations of SAK, an HPLC method was developed (Figures S5 and S6, Supporting Information) and validated for application in plasma protein binding and microsomal metabolism assays. The method presented a lower limit of quantification of 50 ng in 0.1 mL of plasma (Table 1), corresponding to a 10-fold increase in sensitivity compared with previous reports.^24^ After liquid–liquid extraction, the recovery of SAK from rat plasma was approximately 80%, in agreement with values previously described for rat serum and urine.^26^ Plasma protein binding assays revealed extensive binding, with only 8.3 ± 0.1% of SAK remaining in the unbound form, likely due to strong interactions with albumin, which exhibits high affinity for acidic compounds such as SAK. However, elevated plasma protein binding is not necessarily a limitation for drug development, since this interaction is dynamic and reversible. In fact, several clinically used drugs, including diclofenac, ibuprofen, and losartan, display plasma protein binding values higher than 99%.^27–29^

To further investigate the metabolic profile of SAK, the validated HPLC assay was applied in a substrate depletion study using UDPGA and rat liver microsomes (Figure S6, Supporting Information). Previous studies demonstrated that, in rats, the UDPGA-mediated intrinsic clearance (CL_int_) of SAK in microsomes is approximately 4.5-fold higher than that mediated by NADPH.^20^ A predominance of glucuronidation over oxidative metabolism has also been reported in mouse and human microsomes, whereas the opposite profile was observed in dogs.^20^ Based on these findings, the present study focused specifically on UDPGA-mediated metabolism. Our results demonstrated a rapid depletion of SAK, most likely due to glucuronidation, which is consistent with the presence of two phenolic hydroxyl groups in its structure. Taken together with the *in silico* and *in vitro* ADME data—including compliance with Lipinski’s rules, absence of PAINS alerts, satisfactory HPLC recovery, and predictable metabolic behavior—these findings further support the translational potential of SAK as a promising orally active antischistosomal candidate.

Overall, the combination of ADME profiling, toxicity evaluation, and *in vivo* efficacy data positions SAK among the most promising natural flavonoids investigated against *S. mansoni* to date. Although SAK displays extensive plasma protein binding and is susceptible to glucuronidation, these pharmacokinetic characteristics are commonly observed among orally active flavonoids and do not necessarily compromise its therapeutic applicability. Future investigations should focus on pharmacokinetic optimization, assessment of potential synergistic effects in combination with praziquantel (PZQ), and evaluation in chronic schistosomiasis models to further support its development as a novel antischistosomal agent. In conclusion, SAK exhibits favorable drug-like characteristics for oral administration, fulfilling Lipinski’s criteria and showing no PAINS-related structural alerts. The validated analytical method demonstrated efficient recovery from biological matrices and reliable HPLC detection. Although SAK displays high plasma protein binding and undergoes metabolic biotransformation, these properties are consistent with the pharmacokinetic behavior commonly associated with flavonoids. Importantly, SAK showed pronounced antischistosomal activity in a murine model, producing reductions in worm and egg burdens comparable to praziquantel and superior to those reported for other flavonoids evaluated *in vivo*. Furthermore, the absence of detectable toxicity in mammalian cells and *C. elegans* reinforces its favorable safety profile. Taken together, these results highlight SAK as a promising natural product-derived lead compound for the development of new oral therapies against schistosomiasis.

## Materials and Methods

### Reagents and Equipment

Silica gel 60 (63–210 mesh; Merck, Rahway, NJ, USA) and Sephadex LH-20 (GE Healthcare, Chicago, IL, USA) were employed for column chromatography, whereas silica gel F254 plates (Macherey-Nagel, Düren, NRW, Germany) were used for analytical TLC analyses. ^1^H and ^13^C NMR spectra were acquired on a Bruker Ascend Evo 600 spectrometer (Bruker, Billerica, MA, USA), operating at 600 and 150 MHz, respectively, using CD_3_OD (Sigma-Aldrich, St. Louis, MO, USA) as solvent and internal reference. Quercetin (QUE, ≥99% purity), used as the internal standard (IS), as well as nicotinamide adenine dinucleotide phosphate reduced tetrasodium salt hydrate (NADPH) and uridine diphosphoglucuronic acid trisodium salt (UDPGA), were obtained from Sigma-Aldrich (Oakville, ON, Canada). HPLC-grade acetonitrile (ACN) and methanol (MeOH) were purchased from Caledon Laboratories Ltd. (Georgetown, ON, Canada). HPLC analyses were performed using a Shimadzu system composed of a CBM-20A system controller, DGU-20A3R degasser, SIL-20A HT autosampler, LC-20ADVP binary pumps, and CTO-20AC column oven (Shimadzu, Kyoto, Japan). Chromatographic separation was achieved using a Phenomenex Luna 5 μm C_18_ column (250 mm × 4.60 mm; Torrance, CA, USA) coupled to a SecurityGuardTM analytical guard cartridge (Phenomenex, Torrance, CA, USA). The mobile phase consisted of ACN/H_2_O (45:55) containing 0.5% formic acid, delivered at a flow rate of 1.0 mL/min. Autosampler and column temperatures were maintained at 4 and 25 °C, respectively. Detection was carried out in photodiode array (PDA) mode, monitoring SAK and QUE at 285 and 372 nm, respectively. Data acquisition and chromatographic processing were performed using LabSolutions software (version 5.91, Shimadzu, Kyoto, Japan). Ultrafiltration Centrifree devices (Merck Millipore Ltd., Oakville, ON, Canada) were used in plasma protein binding experiments. All additional laboratory reagents and solvents were purchased from Fisher Scientific (Ottawa, ON, Canada) and Labsynth (Diadema, SP, Brazil) and used without further purification.

### Biological Materials

*S. mansoni* (BH strain) was maintained through successive passages in *Biomphalaria glabrata* snails and Swiss mice at the *Núcleo de Pesquisa em Doenças Negligenciadas* (NPDN, Guarulhos University, SP, Brazil). Cercariae obtained from infected snails were used for experimental infections.^28^ Vero cells (African green monkey kidney, ATCC) were cultured in Dulbecco’s Modified Eagle Medium (DMEM) supplemented with 10% heat-inactivated fetal calf serum and 2 mM l-glutamine (Vitrocell, Campinas, SP, Brazil) under standard culture conditions (37 °C, 5% CO_2_).^30^ The nematode *C. elegans* (strain N2) was maintained at 22 °C on nematode growth medium (NGM) agar plates seeded with *Escherichia coli* OP50 according to established protocols.^31^

### Plant Material

Leaves of *B. lateralis* Baker (Asteraceae) were collected in May 2015 in Campos do Jordão, São Paulo State, Brazil (22°42′38.7″S, 45°35′10.1″W). The plant material was identified, and a voucher specimen was deposited in the SPF Herbarium of the *Instituto de Botânica de São Paulo* under accession number 220669. The study was also registered in the Brazilian National System for the Management of Genetic Heritage and Associated Traditional Knowledge (SisGen) under registration number A4123E4.

### Isolation and Characterization

Dried leaves of *B. lateralis* were initially defatted with hexane and subsequently extracted with MeOH. The resulting MeOH extract was partitioned between hexane (0.5 g) and CH_2_Cl_2_ (10.7 g). A portion of the CH_2_Cl_2_ fraction (10.0 g) was subjected to silica gel column chromatography and eluted with mixtures of hexane/EtOAc (9:1, 8:2, 7:3, 1:1, and 3:7), yielding five groups (A–E). Group B (977 mg) was further purified by Sephadex LH-20 column chromatography using hexane/CH_2_Cl_2_ (1:4), CH_2_Cl_2_/acetone (3:2 and 1:4), and pure acetone as eluents, affording 618 mg of sakuranetin (SAK).

### *In Silico* Analysis

Physicochemical properties and pharmacokinetic parameters of SAK were predicted using the SwissADME platform (http://www.swissadme.ch/). As summarized in Table S1 (Supporting Information), the evaluated parameters included molecular weight (MW), number of heavy atoms (NHA), fraction of sp^3^ carbons (Fsp^3^), number of rotatable bonds (NRB), hydrogen bond acceptors (HBA), hydrogen bond donors (HBD), molar refractivity (MR), topological polar surface area (TPSA), and the octanol/water partition coefficient (ClogP). Additional *in silico* predictions included gastrointestinal (GI) absorption, blood–brain barrier (BBB) permeability, cytochrome P450 inhibition profile (including CYP1A2), and the presence of pan-assay interference substructures (PAINS).^19^

### Bioassay Procedures

#### *In* *Vivo* Antischistosomal Activity Evaluation

Female Swiss mice (18–22 g, 3 weeks old; AniLab, Paulínia, SP, Brazil) were subcutaneously infected with 80 *S. mansoni* cercariae and maintained under controlled environmental conditions (24 °C, 12 h light/dark cycle) with *ad libitum* access to food and water. 49 days after infection, animals were randomly allocated into groups of five and treated orally by gavage. SAK was administered as a single oral dose of 400 mg/kg, prepared in 2% EtOH in water (v/v).^32^ The positive control group received praziquantel (PZQ, 400 mg/kg, p.o.), whereas the negative control group received vehicle only. Fourteen days after treatment, mice were euthanized by CO_2_ inhalation, and adult schistosomes were recovered from the hepatic portal system and mesenteric veins using the perfusion technique.^33^ Parasites were counted and separated by sex to determine worm burden reduction (WBR).^34^

#### Randomization and Blinding

To minimize experimental bias, mice were randomly assigned to treatment groups, and the order of euthanasia was also randomized. Although investigators were aware of the treatment allocation, worm burden determination, fecal egg counts, and oogram analyses were independently performed by different researchers. In addition, data analysis was conducted by two investigators who were not directly involved in the experimental procedures.^35^

#### *In Vitro* and *In Vivo* Cytotoxicity

Vero cells were seeded into 96-well plates at a density of 1 × 10^4^ cells per well and incubated for 24 h at 37 °C under 5% CO_2_. Subsequently, cells were exposed to SAK at concentrations ranging from 62.5 to 500 μM for 72 h. Cell viability was determined using the MTT assay, and absorbance was measured at 595 nm with an Epoch Microplate Spectrophotometer (BioTek Instruments, USA). All experiments were carried out in triplicate and independently repeated three times. Results were expressed as percentage cell viability relative to untreated control cells.^36^ The toxicity of SAK was additionally evaluated using *C. elegans* (strain N2) following previously established procedures.^37^ Approximately 25 L4-stage worms per well were transferred to 96-well plates containing a mixture of 60% M9 buffer, 10 μg/mL cholesterol, and 40% BHI medium. SAK was tested at concentrations ranging from 100 to 1000 μM. After incubation for 24 h at 22 °C, worm viability was assessed microscopically based on morphology and motility using an AE2000 inverted microscope (Motic, Canada). Nonmotile and rigid nematodes were considered dead. Each experiment was performed in triplicate and independently repeated three times.

### HPLC Assay Development

#### Extraction Procedure

Aliquots of SAK stock solutions prepared in MeOH were added to 100 μL of rat plasma at different concentrations, followed by the addition of 10 μL of internal standard (IS, 1000 ng), 100 μL of HCl (1 mol/L), and 500 μL of EtOAc. Samples were vortex-mixed for 30 s and centrifuged at 3000*g* for 10 min. The organic phase was collected and evaporated under reduced pressure. The resulting residues were reconstituted in 100 μL of MeOH, and 50 μL of each sample was injected into the HPLC system. Recovery was determined by comparing the peak areas obtained from plasma-extracted SAK with those of nonextracted standard solutions.

#### Method Validation

Method development and validation were conducted in accordance with the guidelines established by the Brazilian National Health Surveillance Agency (ANVISA),^38^ the European Medicines Agency (EMA),^39^ and the United States Food and Drug Administration (FDA).^40^ Primary stock solutions of sakuranetin (SAK) and quercetin (QUE) were individually prepared in MeOH at a concentration of 1 mg/mL. Working standard solutions were freshly prepared each day by serial dilution of the stock solutions with MeOH, and all stock solutions were stored under refrigeration between uses. Calibration standards were prepared by spiking 100 μL of rat plasma with SAK at concentrations of 25, 50, 100, 250, 1000, 2500, 5000, and 10,000 ng/mL, together with QUE at a fixed concentration of 1000 ng/mL, followed by the extraction procedure described above.

#### Calibration, Linearity, Accuracy, and Precision

The use of rat-derived biological specimens in these experiments was approved by the University of Alberta Health Sciences Animal Care and Use Committees (ACUCs). All plasma samples were obtained from male Sprague–Dawley rats. Drug-free plasma was used as a blank matrix to verify the absence of interference from endogenous components with the chromatographic elution of SAK and the internal standard (IS), quercetin (QUE). For each analytical run, calibration curves were prepared by spiking 100 μL of rat plasma with eight concentrations of SAK (25, 50, 100, 250, 1000, 2500, 5000, and 10,000 ng/mL). The lowest concentration corresponded to half of the lower limit that satisfied the acceptable validation criteria, while the highest concentration exceeded the upper validation range. A fixed concentration of the internal standard (IS, 1000 ng/mL) was added to all samples. Method linearity was evaluated using three independent calibration curves containing the eight nominal concentrations of SAK. Weighted linear regression (1/concentration^2^) of the peak area ratio between SAK and QUE versus nominal SAK concentration was applied to determine the slope, intercept, and correlation coefficient (*r* ^2^). Method precision and accuracy were assessed using a minimum of five replicates for each concentration level and sample volume, including the lower limit of quantification (LLOQ), low-quality control (LQC), medium-quality control (MQC), and high-quality control (HQC) samples. The LLOQ was defined as the lowest concentration that met acceptable criteria for accuracy and precision, within a deviation range of 20%. Blank samples (plasma without SAK or IS) were included in each analytical run. Intraday accuracy and precision were determined based on the analyzed SAK concentrations, and the assay was fully validated using 100 μL plasma samples over three independent days. Precision was expressed as the coefficient of variation (CV%), whereas accuracy was calculated as the mean percentage error between measured and nominal concentrations.^41^

#### Recovery and Carryover

The recovery of SAK and QUE following liquid–liquid extraction was evaluated by comparing the mean peak area ratios of plasma samples spiked prior to extraction with those of plasma extracts spiked with the analytes after extraction at five concentration levels. Carryover was assessed by monitoring the presence of chromatographic peaks in blank samples injected immediately after the highest calibration standard.

#### Selectivity and Sensitivity

Blank rat plasma samples were independently prepared and used as blank calibrators to evaluate potential interference from endogenous plasma components. The lower limit of quantification (LLOQ) was defined as the lowest concentration on the calibration curve that met acceptable criteria for precision and accuracy (±20%). In addition, analyte responses at the LLOQ were required to be at least 5-fold higher than the responses observed in analyte-free calibrator samples.^40^

#### Stability

The stability of SAK was evaluated in rat plasma under conditions representative of sample collection, storage, processing, and analysis. Stability assays included: (a) storage of SAK-spiked plasma samples at −80 °C for 1 and 7 days prior to extraction; (b) storage at room temperature for 4 and 24 h before extraction; (c) storage at 4 °C for 4 and 24 h before extraction; (d) freeze–thaw stability by thawing samples from −20 to 22 °C prior to extraction; and (e) postextraction stability under autosampler conditions, in which extracted and reconstituted samples were maintained at 4 °C for 4 and 20 h. Stability was assessed by comparing analyte responses obtained from stability samples with those from freshly prepared plasma standards at equivalent concentrations. During handling outside freezer conditions, all tubes were protected from light using aluminum foil and maintained under reduced light exposure by turning off fluorescent laboratory lighting.

#### Plasma Protein Binding

Plasma protein binding was determined by ultrafiltration using Centrifree devices (Merck Millipore Ltd., Oakville, ON, Canada). Briefly, 1000 μL of plasma was spiked with SAK at a final concentration of 10,000 ng/mL and incubated in a shaking water bath at 37 °C for 1 h. After incubation, a 100 μL aliquot of plasma was directly transferred to clean tubes, spiked with the internal standard (IS), and analyzed to determine total SAK concentration. The remaining 900 μL was transferred to the ultrafiltration device and centrifuged at 2000*g* for 1 h at 37 °C. The resulting filtrates were subsequently spiked with IS and analyzed for determination of the unbound SAK concentration. All experiments were performed in duplicate, and plasma protein binding was calculated using the following equation

$$unbound\;fraction\;\left(\%\right)=\frac{filtrate\;concentration}{plasma\;concentration}\times 100$$

#### Phase II Metabolism

The validated assay was applied to investigate the glucuronidation pathway of SAK using rat liver microsomes obtained from male Sprague–Dawley rats maintained on a standard diet. Microsomal preparations were derived from a previous study evaluating the effects of diet on bupivacaine metabolism. After preparation, microsomal samples were immediately frozen in liquid nitrogen and stored at −80 °C until analysis. SAK (1000 ng/mL) was incubated in a shaking water bath (37 °C, 50 rpm) with liver microsomes containing 1 mg/mL microsomal protein in the presence of 5 mM magnesium chloride hexahydrate dissolved in 0.5 mM potassium phosphate buffer (pH 7.4), in a final reaction volume of 1 mL. The reaction was initiated by the addition of 2 mM uridine diphosphate glucuronic acid (UDPGA) following a 5 min pre-equilibration period. Aliquots (100 μL) were collected at 0, 15, 30, 45, 60, and 120 min, and the reaction was terminated by the addition of 100 μL of HCl (1 mol/L). Subsequently, the internal standard was added, followed by the extraction procedure described in Section 2.6.1 and HPLC analysis to quantify the remaining concentration of SAK. The half-life (*t*_1/2_) was determined from the slope of the log-transformed percentage of SAK remaining over time. Intrinsic clearance (CL_int_) for microsomal glucuronidation was calculated from the substrate depletion curve as

${\mathrm{CL}}_{\mathrm{int}}=\frac{0.693}{t_{1/3}\times \left[M\right]}$ , where *M* corresponds to the microsomal protein concentration.^42^

### Statistical Analysis

Data are expressed as mean ± standard deviation (SD). Statistical analyses were conducted using GraphPad Prism version 10.0 (GraphPad Software, San Diego, CA, USA). Differences among groups were evaluated by one-way analysis of variance (ANOVA) followed by Tukey’s post hoc test, with statistical significance established at *p* < 0.05.

### Ethics Statement

Animal experiments were conducted in accordance with the ARRIVE guidelines established by the National Centre for the Replacement, Refinement, and Reduction of Animals in Research (NC3Rs). All procedures complied with the Brazilian Guidelines for the Care and Use of Laboratory Animals (Law No. 11,790/2008). The experimental protocol was approved by the *Comissão de Ética no Uso de Animais* (CEUA), Brazil (protocol no. 065/24). In addition, the protocol related to the procurement of rat liver microsomes was approved by the University of Alberta Health Sciences Animal Care and Use Committees (ACUCs).

## Supplementary Material



## Notes

^1^H NMR spectrum of SAK (δ, 600 MHz, CD_3_OD) (Figure S1); ^13^C NMR spectrum of SAK (δ, 150 MHz, CD_3_OD) (Figure S2); DEPT NMR spectrum of SAK (δ, 150 MHz, CD_3_OD) (Figure S3); ESI-HRMS spectrum (positive mode) of SAK (Figure S4); HPLC analysis and UV spectra obtained to QUE and SAK at 285 and 372 nm (Figure S5); Mean ± SD of the percent decline of SAK in the presence of rat liver microsomes with UPDGA (performed in quadruplicate) (Figure S6); HPLC analysis of glucuronidation of SAK by rat liver microsomes at times 0, 15, 30, 45, 60, and 120 min at 285 nm (Figure S7); physicochemical, pharmacokinetic, and drug-likeness in silico prediction for SAK (Table S1); stability of SAK in rat plasma under different conditions (*n* = 3) (Table S2); *in vitro* antiparasitic activity and toxicity profile of SAK (Table S3) (PDF)
