# Supplementary material for: A Multidimensional Evaluation of Sakuranetin against Schistosoma mansoni: From Drug-Likeness to In Vivo Efficacy
Source: ACS Omega. 2026 Jun 12;11(25):38125–33. doi: 10.1021/acsomega.6c03820 (PMC13325134; doi:10.1021/acsomega.6c03820)
Supplement: Supplementary file 1 [file ao6c03820_si_001.pdf]

A multidimensional evaluation of sakuranetin against *Schistosoma mansoni* - from drug-likeness to *in vivo* efficacy and metabolization

Dalete Christine S. Souza[1,2], Pedro Enrico H. Tesser[1], Erica Fernanda da S. Tirelli[1], Ruqaya Jasim[2], Monique C. Amaro[3], Rayssa A. Cajas[3], Dion R. Brocks[2], Josué de Moraes[3,4], João Henrique G. Lago[1]

[1]Center for Natural and Human Sciences, Federal University of the ABC, São Paulo, 09210-180, SP, Brazil.

[2]Katz Group Centre for Research, University of Alberta, Edmonton, Alberta, T6G 2G5, Canada.

[3]Center for Research on Neglected Diseases, Guarulhos University, São Paulo, 07023-070, Brazil.

[4]Centre for Research on Neglected Diseases, Brazil University, São Paulo, 08230-030, Brazil.

SUPPORTING INFORMATION

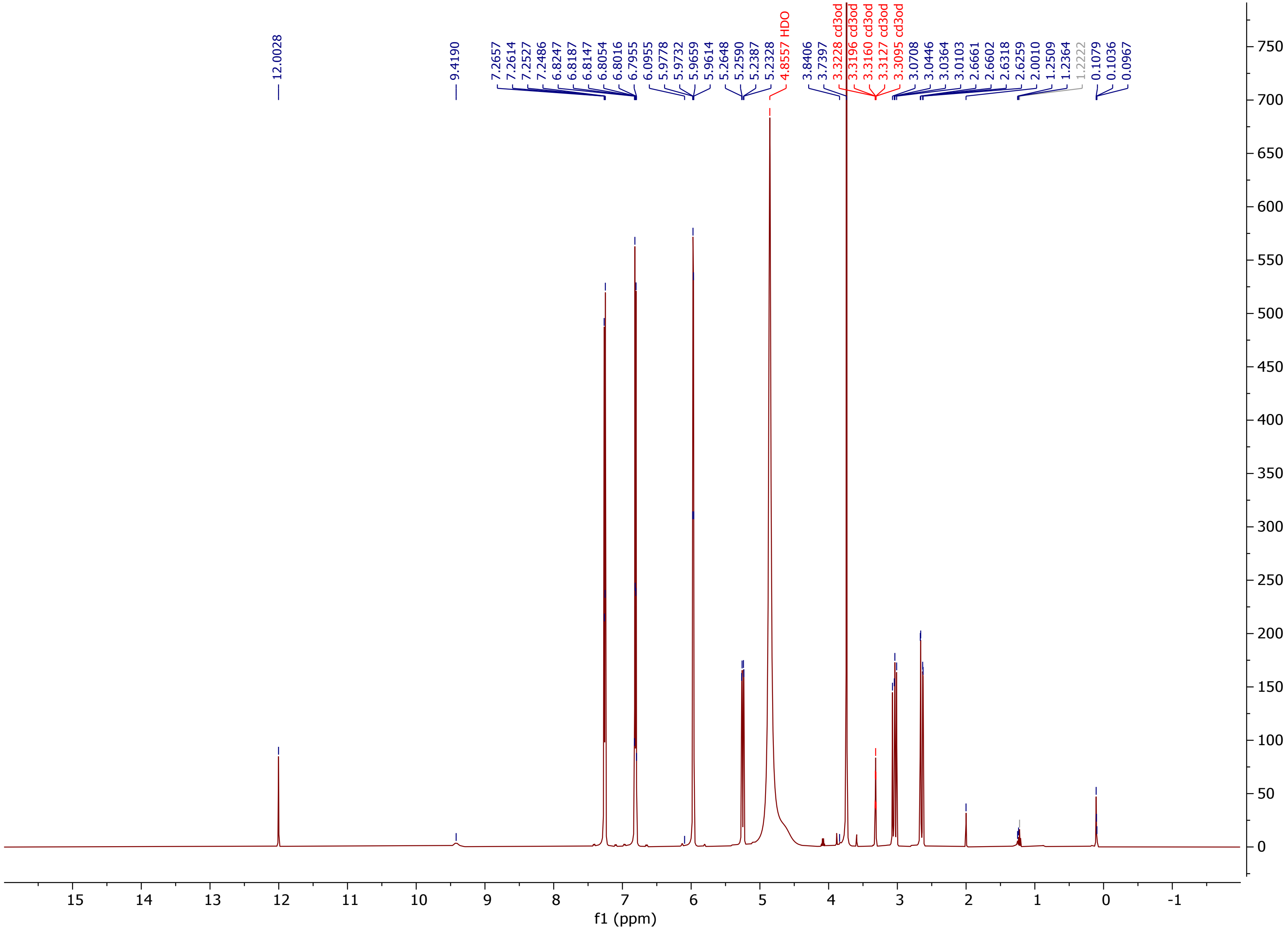

Figure S1. ^{1}H NMR spectrum of SAK (δ, 600 MHz, CD_3OD)

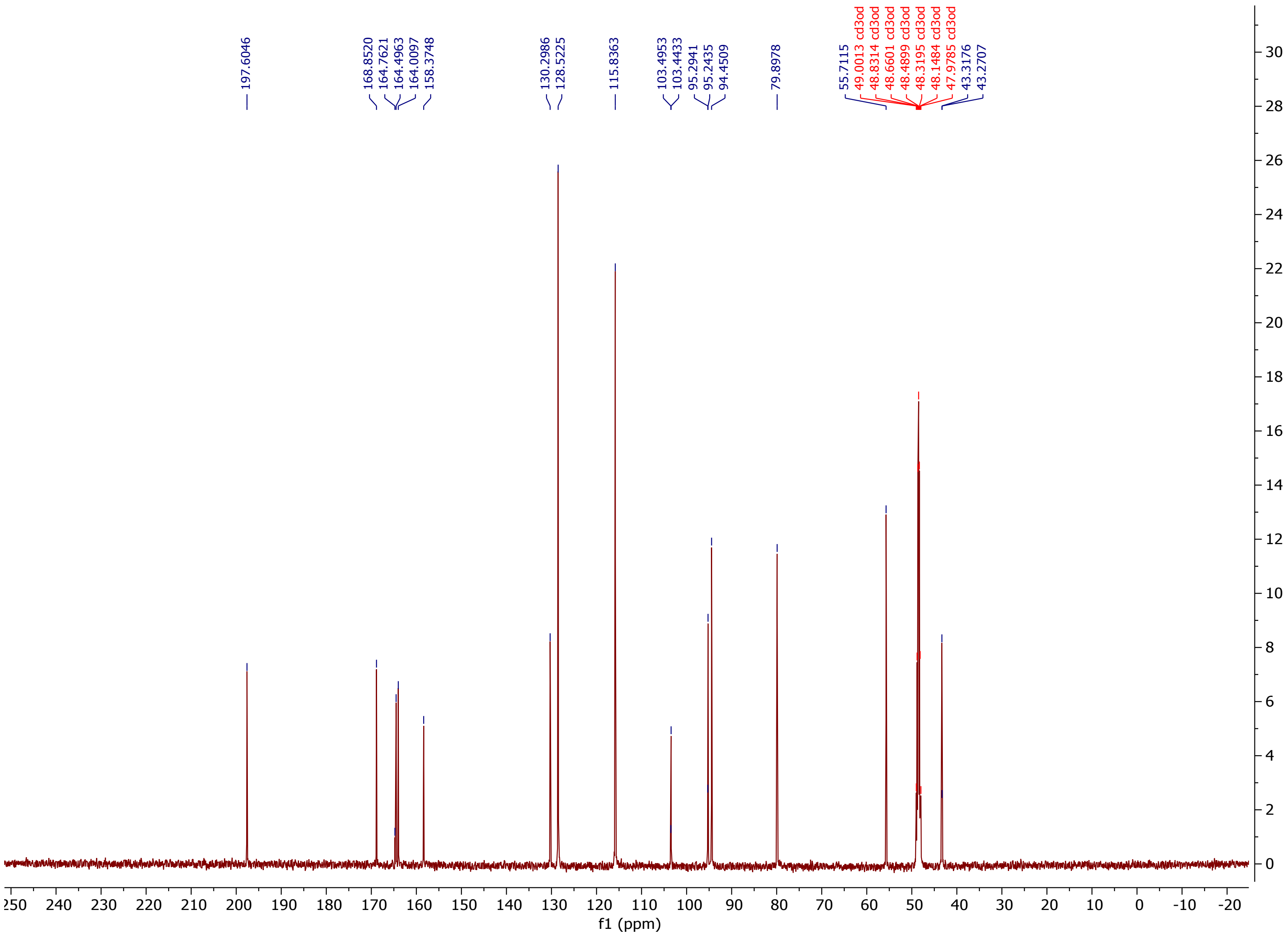

Figure S2. ^{13}C NMR spectrum of SAK (δ, 150 MHz, CD_3OD)

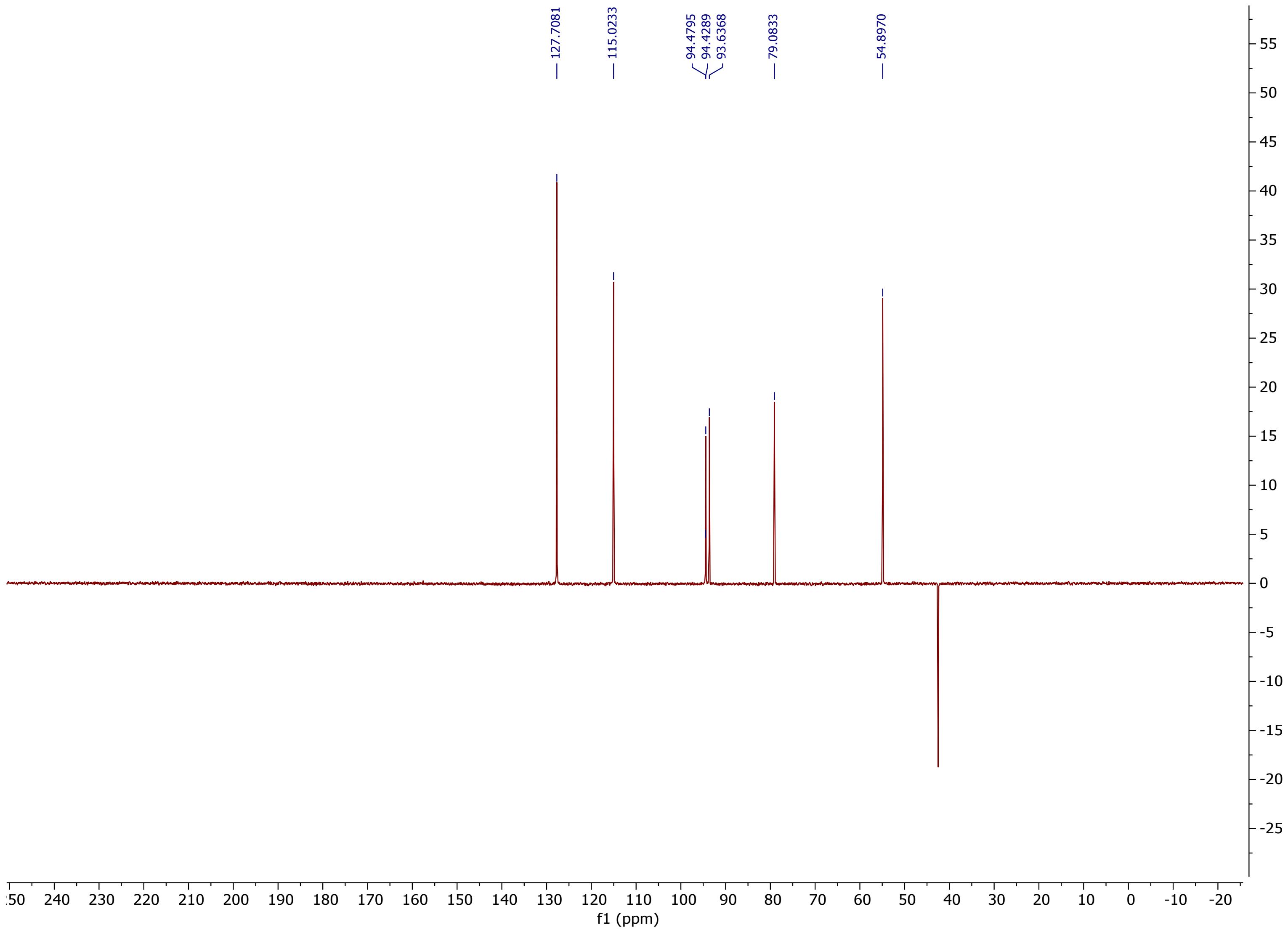

Figure S3. DEPT NMR spectrum of SAK (δ, 150 MHz, CD_3OD)

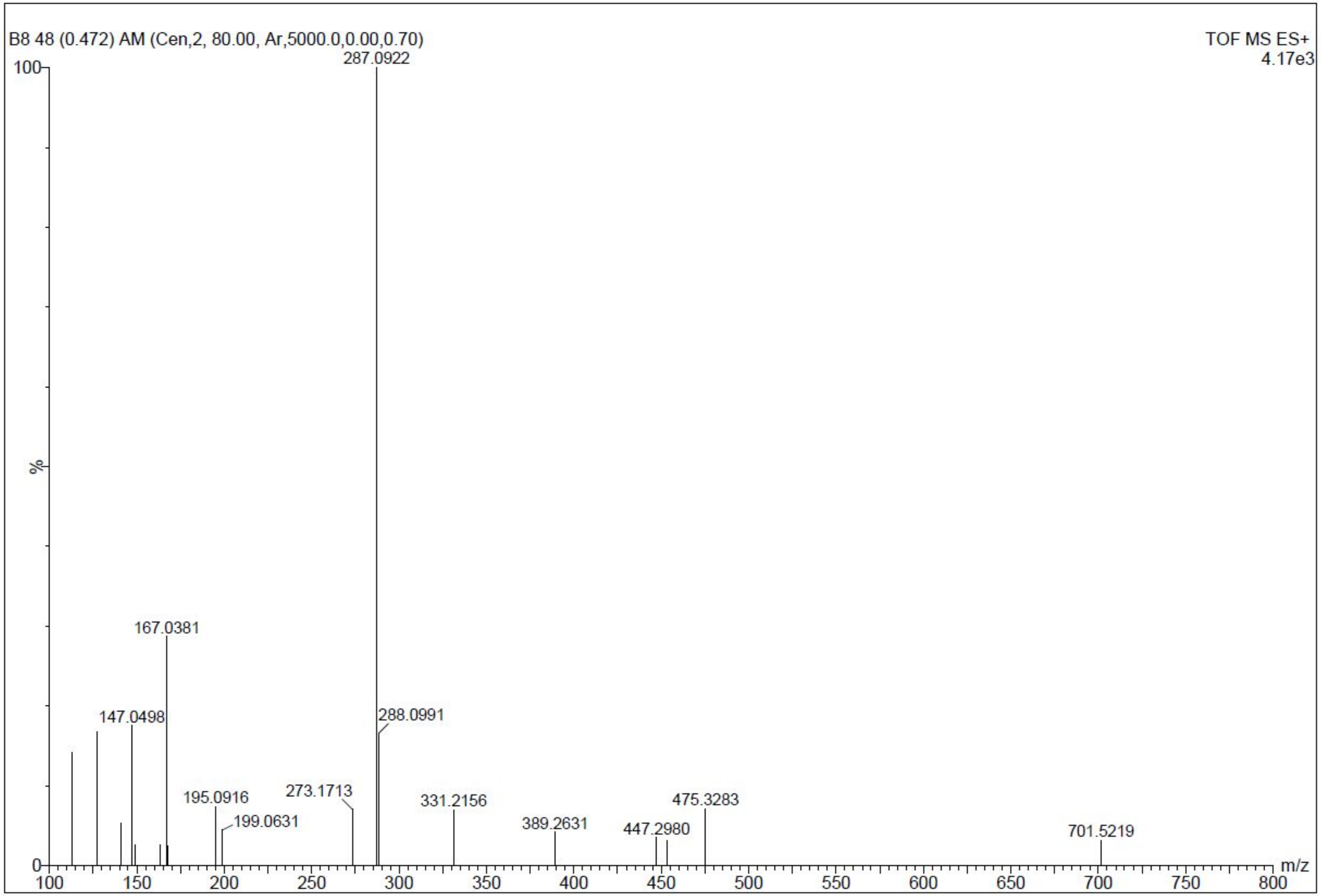

Figure S4. ESI-HRMS spectrum (positive mode) of SAK

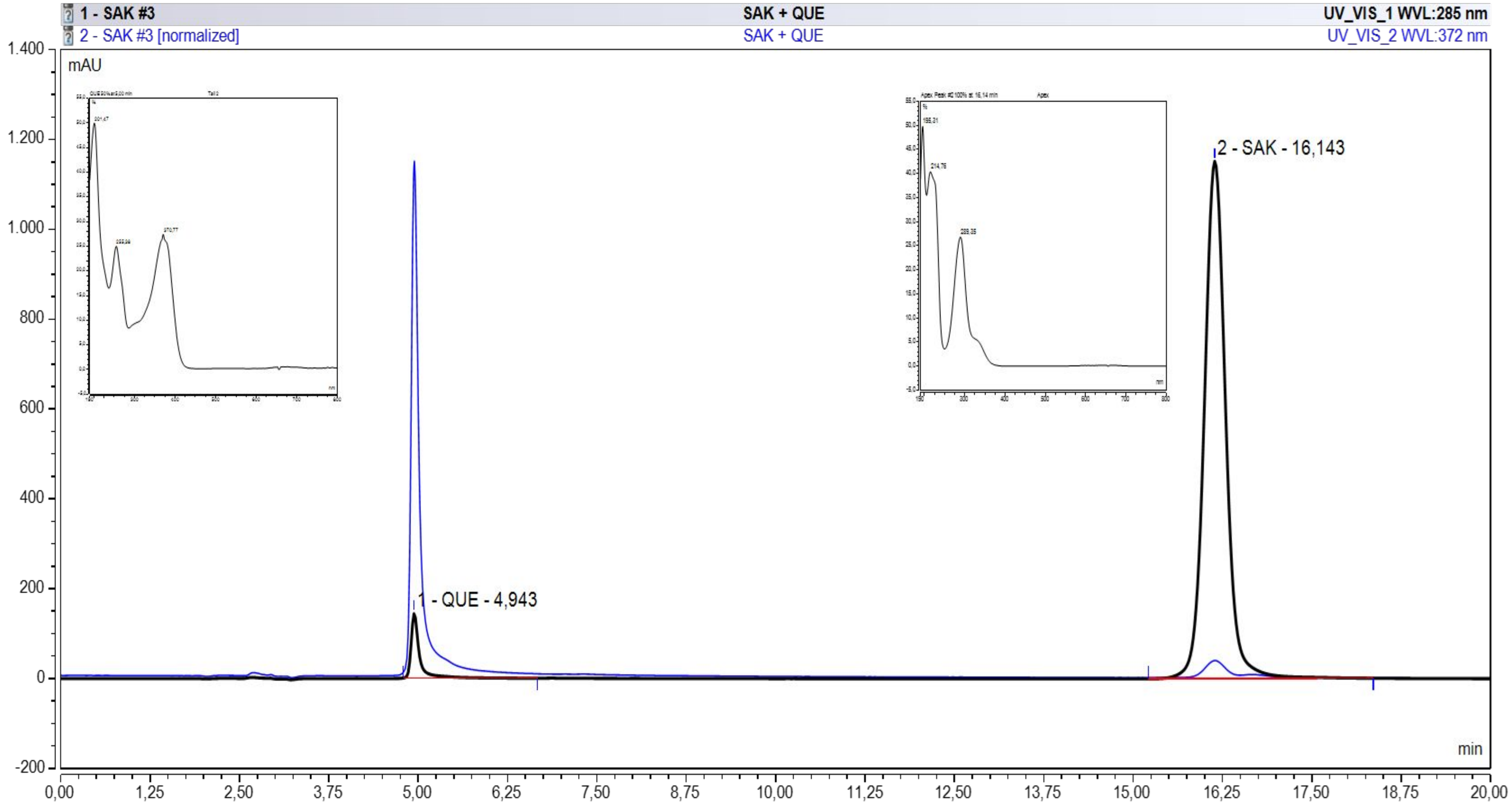

Figure S5. HPLC analysis and UV spectra obtained to QUE and SAK at 285 and 372 nm. The mobile phase was composed by ACN:H_2O 45:55 with 0.5 % formic acid at flow rate of 1.0 mL/min during 20 min.

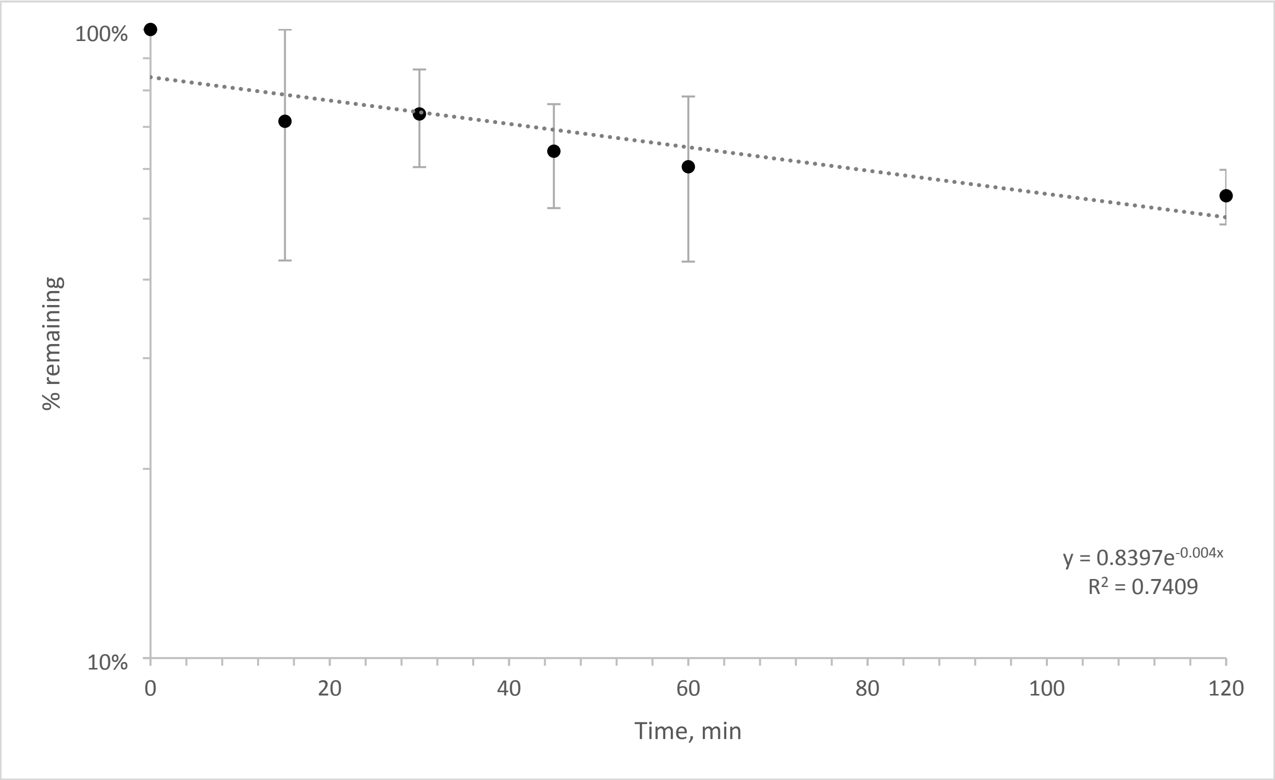

Figure S6. Mean ± SD of the percent decline of SAK in the presence of rat liver microsomes with UPDGA (performed in quadruplicate). The dashed line represents the exponential regression of the mean decline vs. time relationship. The decline was significant with inclusion of all of the individual data points of the three incubations ($p < 0.05$). The mean r^2 of the four runs were 0.7198.

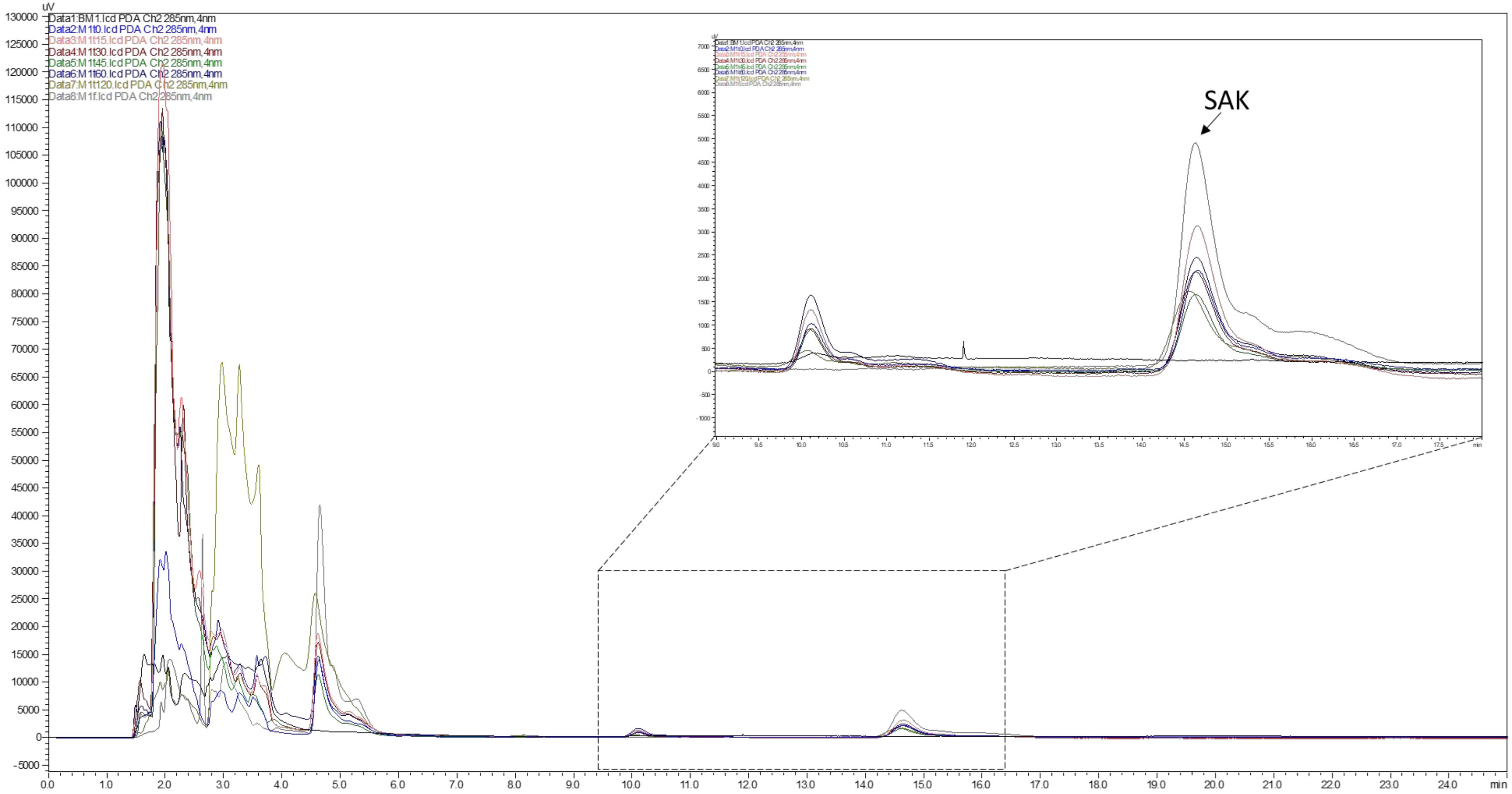

Figure S7. HPLC analysis of glucuronidation of SAK by rat liver microsomes at times 0, 15, 30, 45, 60, and 120 minutes at 285 nm. The mobile phase was composed by ACN:H_2O 45:55 with 0.5 % formic acid at flow rate of 1.0 mL/min during 25 min.

Table S1. Physicochemical, pharmacokinetic and drug-likeness *in silico* prediction for SAK.

**SAK**							
**Physicochemical Properties**		**Lipophilicity**		**Pharmacokinetics**		**Drug likeness**	
Molecular formula	$C_{16}H_{14}O_5$	CLogP	2.25	GI absorption	High	Lipinski	Yes
MW (g/mol)	286.28	**Water Solubility**		BBB permeant	Yes	Ghose	Yes
NHA	21	Solubility	Moderately soluble	P-gp substrate	No	Veber	Yes
F Csp^3	0.19	**Medicinal Chemistry**		CYP1A2 inhibitor	Yes	Egan	Yes
NRB	2	PAINS	No	CYP2C19 inhibitor	Yes	Muegge	Yes
HBA	5	Brenk	No	CYP2C9 inhibitor	No	Bioavailability Score	0.55
HBD	2	Lead likeness	Yes	CYP2D6 inhibitor	No		
MR	76.04	Synthetic Accessibility	3.11	CYP3A4 inhibitor	Yes		
TPSA (Å^2)	75.99			Skin permeation (cm/s)	-6.02		

MW – molecular weight; NHA – number of heavy atoms; Fsp^3 - unsaturation index; NRB – number of rotatable bonds; HBA - hydrogen bond acceptor groups; HBD - hydrogen bond donor groups; MR – molar refractivity; TPSA - topological polar surface area; ClogP - logarithm of *n*-octanol/water; GI - gastrointestinal absorption; BBB - blood-brain barrier penetration; CYP1A2 - cytochrome P450 family 1 subfamily A member 2, involved in the metabolism of xenobiotics; PAINS - pan-assay interference sub-structures.

Table S2. Stability of SAK in rat plasma under different conditions (*n* = 3).

Conditions	Concentration (ng/mL)	Mean Peak Height	% Difference
Zero Time	500	1118	-
	7500	15693	-
Autosampler Conditions at 4ºC, 4h	500	1213	8.50
	7500	16534	5.36
Autosampler Conditions at 4ºC, 20h	500	1034	-7.52
	7500	16605	5.81
Benchtop, 4h	500	1197	7.07
	7500	16208	3.28
Benchtop, 24h	500	1297	16.05
	7500	15749	0.36
Fridge at 4ºC, 4h	500	1201	7.46
	7500	15290	-2.57
Fridge at 4ºC, 24h	500	1316	17.75
	7500	14980	-4.54
Freezer at -80ºC, 24h	500	1102	-1.37
	7500	16451	4.83
Freezer at -80ºC, 7 days	500	1187	6.23
	7500	14808	-5.64
Freeze & thaw from -20ºC to 22ºC	500	1304	16.64
	7500	16594	5.74

Table S3. *In vitro* antiparasitic activity and toxicity profile of SAK.

Assay	Model/System	Endpoint	Value (μM)	Interpretation
Antiparasitic activity	*Schistosoma mansoni* (adult worms)	EC_{50}	> 50	No detectable activity within tested range
Cytotoxicity	Vero cells	CC_{50}	> 500	No cytotoxicity observed
In vivo toxicity surrogate	*Caenorhabditis elegans* (L4 stage)	LC_{50}	> 1000	No acute toxicity observed

Antiparasitic activity was evaluated against adult *Schistosoma mansoni* worms, with no detectable activity observed at concentrations up to 50 μM. Cytotoxicity was assessed in Vero cells using the MTT assay after 72 h of exposure. Toxicity in *Caenorhabditis elegans* was evaluated based on motility and morphology after 24 h of exposure. Values represent the highest concentrations tested in each assay.
